# Estimation of Stroke's Motor Function Ability Using Multimodal Biomarkers and the Role of Contralesional Motor Area

**DOI:** 10.1002/brb3.70492

**Published:** 2025-05-08

**Authors:** Da‐Hye Kim, Gyu Hyun Kwon, Seong‐Whan Lee, Laehyun Kim

**Affiliations:** ^1^ Bionics Research Center Korea Institute of Science and Technology Seoul Republic of Korea; ^2^ Graduate School of Technology and Innovation Management Hanyang University Seoul Republic of Korea; ^3^ Department of Brain and Cognitive Engineering Korea University Seoul Republic of Korea; ^4^ Department of HY‐KIST Bio‐Convergence Hanyang University Seoul Republic of Korea; ^5^ Department of Artificial Intelligence Korea University Seoul Republic of Korea

**Keywords:** biomarker, contralesional motor area, motor tasks, rehabilitation, stroke

## Abstract

**Purpose:**

In the chronic phase, many stroke survivors did not regain their pre‐stroke upper limb movement capabilities. This emphasizes the crucial role of assessing motor function in patients with stroke, as it provides valuable insights into setting effective rehabilitation goals. Accordingly, this study aimed to investigate the electroencephalography (EEG)‐based functional brain network properties in stroke patients during motor tasks and assess their utility in predicting the upper limb Fugl‐Meyer Assessment (UL‐FMA) scores.

**Methods:**

We performed a comparative analysis of brain properties, including EEG power and network characteristics, in stroke patients and a healthy control (HC) group. Also, we selected prognostic factors of brain properties during voluntary movement for patients' motor function ability using stepwise regression analysis.

**Findings:**

Stroke patients manifested reduced global efficiency relative to the HC group, signifying impaired information processing attributed to brain injury. Local analyses highlighted pronounced disparities in the contralesional motor area (MA) between stroke patients and the HC group, revealing patterns indicative of compensatory mechanisms. Leveraging a multimodal approach incorporating EEG power and network features within the contralesional MA yielded a robust model for motor function estimation, outperforming unimodal models (adjusted R^2^ 0.99, RMSE 0.13). The findings of this study outperformed other models for estimating the motor abilities of chronic stroke patients. Another chronic stroke dataset was used to externally validate this study, and it had an adjusted R^2^ of 0.95. This suggests that the results of this study can be generalized.

**Conclusion:**

Our findings provide insight into the brain properties of stroke‐related motor impairment. These results underscore the pivotal role of the contralesional MA in assessing UL‐FMA scores and represent how a multimodal approach to this area can suggest the possibility of using it as a meaningful biomarker for motor function. They also have potential implications for the development of individualized rehabilitation strategies, particularly during the chronic phase of recovery.

## Graphical abstract

This study reports that the contralesional MA plays a key role in motor function estimation, serving as a potential biomarker for stroke‐related motor impairment. This approach can aid in developing personalized rehabilitation strategies, particularly during the chronic recovery phase.

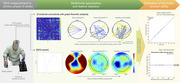

## 1 Introduction

Stroke ranks approximately as a global arbiter of long‐term disability, representing significant challenges in the recovery of motor function—a fundamental in the rehabilitation of survivors. While a quarter of patients regain full motor function, the remainder face up to an enduring battle with motor deficits; a mere 35% recover their pre‐stroke upper limb capabilities in the chronic phase after stroke (Dobkin 2005). Also, over half of these stroke survivors grapple with impaired hand motor function, a critical determinant of daily independence and quality of life (Nowak 2008). Hence, it is imperative to investigate the cerebral characteristics during motor execution to assess hand motor function even in the stroke chronic phase.

The traditional diagnostic modalities—functional magnetic resonance imaging (fMRI) and positron emission tomography (PET)—have been instrumental in decoding the neuroplasticity changes and pathophysiological foundations of stroke‐induced motor impairment (Dechaumont‐Palacin et al. 2008; Grefkes et al. 2008; He et al. 2013; Honda et al. 1997; Johansen‐Berg et al. 2002; Ward et al. 2006). These modalities, however, have relatively low temporal resolution and are less adept at mapping the dynamic neural interplay during motor tasks. While most studies using fMRI or PET focus on analyzing resting‐state brain activity due to their inherent limitations in temporal resolution, electroencephalography (EEG) presents an effective alternative. Its real‐time monitoring capability and high temporal resolution make it particularly suited for detecting nuanced shifts in brain activity that are pivotal during the rehabilitation period. This difference represents the unique advantages of EEG in exploring dynamic brain functions during motor tasks.

The recovery of motor function in stroke patients is closely linked to brain plasticity and the reorganization of the motor cortex. Various approaches are essential for the analysis of the characteristics associated with impaired motor function following a stroke. Going beyond the typical use of EEG, which focuses primarily on brain activation characteristics, in recent years some researchers have used graph theory analysis to identify characteristics of the brain in stroke patients (de Vico Fallani et al. 2009; Philips et al. 2017). By applying graph‐theoretic analyses to brain data, researchers have begun to find the unique brain network characteristics of stroke survivors during motor tasks, relating these patterns with degrees of motor impairment. de Vico Fallani et al. (2009) compared the efficiency of brain networks between stroke and HC during motor tasks such as finger tapping (de Vico Fallani et al. 2009). They found that stroke had a significantly reduced ability to integrate information compared to HC, suggesting that there were network characteristics of the stroke brain that could not be identified from the activation features. Philips et al. (2017) found that network efficiency in the unaffected hemisphere of stroke patients significantly correlated with patients' upper limb FMA and was associated with functional improvement (Philips et al. 2017). Kim et al. (2015) illustrated that stroke exhibits distinct brain network characteristics across different motor tasks, as detected by EEG (Kim et al. 2015). These characteristics were found to correlate with stroke motor function abilities. This approach has been bolstered by reviews, which have shed light on the efficacy of graph theory metrics in distinguishing the altered functional connectivity of brains affected by stroke from undamaged brains. De Vico Fallani et al. (2014) reviewed the utility of graph theory metrics for comparing task‐related activity between stroke and HC (De Vico Fallani et al. 2014). Taya et al. (2015) conducted a review, focusing on the utility of brain network analysis for comparing pre‐ and post‐task EEG characteristics (Taya et al. 2015). In another review, Jiang et al. (2013) compared the characteristics of functional connectivity of disease‐affected and unaffected brains. Their findings demonstrated the utility of graph‐theoretic analyses for comparing functional connectivity in diseased and normal brains (Jiang et al. 2013). Despite these advancements, a gap remains in translating these observed neural patterns into practical clinical tools for motor function assessment in stroke patients. These previous studies only suggest the possibility that task‐related network analysis correlates with stroke motor function ability but do not provide direct biomarkers for clinical assessment of motor function. To further enhance rehabilitation effectiveness, it is essential to infer a patient's motor function abilities based on these biomarkers via brain activation and networks. These previous studies that represented aspects of unique brain network patterns help to tailor rehabilitation protocols more precisely, potentially improving outcomes by aligning therapies with individual neural profiles. This approach not only enhances the specificity of treatment but also provides a clear framework for evaluating progress through neurophysiological markers.

To address this, our study is predicated on the premise that there are different patterns of information flow between damaged and normal brains (Jiang et al. 2013). Based on findings revealing that pathological interactions between contralesional and ipsilesional motor areas (MA) may serve as a crucial pathophysiological factor contributing to motor impairment in stroke patients (Grefkes and Fink 2011), we assume that through the brain activities, including graph theory, applied to task‐induced EEG data. We can illuminate distinctive brain network characteristics associated with motor function capabilities after a stroke. We anticipate these characteristics have the potential to serve as biomarkers, offering a quantifiable measure of the brain's motor network integrity and efficiency. Our objective is twofold: firstly, to validate the utility of EEG and graph theory in capturing these biomarkers during motor tasks; and secondly, to demonstrate how these biomarkers can be integrated into a prognostic model of stroke motor function. By achieving this, we anticipate not only enriching the understanding of motor function post‐stroke but also pioneering a shift towards precision medicine in stroke rehabilitation. Such a tool would not only advance clinical practice but also significantly enhance the quality of life for stroke survivors by providing more targeted and effective rehabilitation strategies.

## 2 Materials and Methods

### 2.1 Participants

This study included eleven participants diagnosed with chronic unilateral upper limb motor impairment resulting from ischemic stroke (n = 11, mean age 52.8 years). The HC group consisted of age‐matched individuals without motor impairment and brain damage (n = 12, mean age 57.8 years, 4 females). All of the participants were right‐handed and had not taken any medication for the nervous system in the previous two months. For external validation for our result, we got an additional dataset from other stroke patients (n = 10, mean age 56.3 years). This dataset was measured at two different stroke phases: the subacute phase (4 weeks post‐stroke onset ± 2 weeks) and the early chronic phase (13 weeks post‐stroke onset ± 2 weeks). As another chronic dataset, we analyzed this early chronic phase dataset, which had the same experimental protocol as this study and performed grasping movements with their paretic hand (Table 1).

**TABLE 1:** Demographics of study participants with stroke.

	No.	Age	Sex	AH	Months after onset	Motor function ability (AH)		
						UL‐FMA	GS‐palm	GS‐pinch/tip
Chronic phase	1	58	M	Rt.	62.8	31	1.26	0.46
	2	56	F	Lt.	51.3	56	3	1.9
	3	49	M	Lt.	49.0	44	2.3	2
	4	52	F	Rt.	61.8	52	3.5	1.6
	5	53	M	Lt.	61.6	32	0	0
	6	57	M	Rt.	67.3	40	3.43	1.8
	7	46	F	Rt.	15.9	54	3.9	2.13
	8	59	M	Lt.	56.5	48	4.7	5.43
	9	52	M	Lt.	37.3	45	2.73	5.89
	10	41	M	Rt.	52.9	59	5.36	3.63
	11	58	M	Rt.	34.3	63	5.9	5.5
	Mean	52.82	M (72.73%)	Rt. (54.55%)	50.06	47.64	3.28	2.76
	Std.	5.67			15.36	10.46	1.73	2.05
Early chronic phase	1	54	F	Rt.	3.4	51	0.89	2.27
	2	69	M	Rt.	3.87	66	2.74	6.14
	3	31	M	Rt.	3	66	6.38	12.21
	4	52	M	Rt.	3.17	36	0.63	1.75
	5	54	M	Rt.	3.37	61	1.34	4.37
	6	66	M	Rt.	2.97	55	2.39	5.55
	7	62	F	Lt.	2.97	21	1.18	1.46
	8	60	M	Rt.	3.37	46	0.37	1.32
	9	60	M	Rt.	3.8	14	0.06	0.04
	10	55	M	Lt.	3.3	66	1.94	5.03
	Mean	56.3	M (80%)	Rt. (80%)	3.32	48.20	1.79	4.01
	Std.	10.5			0.31	18.95	1.83	3.54

Abbreviations: **AH**, affected hand; **F**, female; **GS**, grip strength; **Lt**, left; **M**, male; **Rt**, right; **Std**., standard error; **UL‐FMA**, upper limb Fugl‐Meyer Assessment.

Inclusion criteria for patients included a survival period of more than one year following ischemic stroke and no prior history of neurological disease. Exclusion criteria applied to patients with pacemakers, claustrophobia, discomfort related to EEG electrode placement, and individuals with cognitive impairments that hindered their comprehension of instructions. Control group participants were recruited through the Samsung Medical Center (SMC) and were matched in terms of age with the stroke patient group.

The SMC recruited all experimental participants, and those with stroke underwent an EEG experiment and motor function tests at the SMC. In addition, the patients’ grip strength, which involves physical strength for grasping movements, was tested to assess their hand‐motor function abilities. They were also tested using the upper limb Fugl‐Meyer Assessment (UL‐FMA), a general upper limb performance‐based impairment index for stroke patients. All the tests were performed on the same day as the EEG experiments. Patients with motor impairments in the moderate (21 to 50 points) or mild (51 to 66 points) categories were included based on their UL‐FMA scores (Table 1). To explore characteristics during motor execution, severe cases unable to move their hands were excluded (Fugl‐Meyer et al. 1975). Stroke lesion locations were categorized by a radiologist using fMRI data. HC participants with any motor and neurological disorders were excluded.

This study received approval from the Institutional Review Boards (IRBs) of both the Korea Institute of Science and Technology (KIST) and SMC (KIST IRB; KIST 2013‐009; SMC IRB; SMC 2013‐02‐091). All experiments were conducted in compliance with IRB guidelines, and written informed consent was obtained from all participants prior to their involvement in the study. In addition, participants gave permission for their experimental data to be published.

### 2.2 Experimental Designs

We analyzed brain network properties during motor tasks in stroke patients, focusing on upper limb motor abilities. Participants performed an active task involving hand motor execution and motor intention. In this study, the robot's only role was to assist with the release movement, allowing the patient to focus only on the grasping movement. Hand grasping of the affected limb is associated with motor performance deficits and is an important element of rehabilitation (Boissy et al. 1999; Duncan 1987). During this task, stroke patients were instructed to firmly grasp a robotic handle with their paretic hand until instructed to release. The researchers encouraged the patients who participated in the study to perform their best effort to enhance grasp execution with their motor intention, even if motor execution was not performed well due to stroke damage. In this study, motor intention is defined as the goal of making an attempt at a movement with volitional movement, regardless of whether the patient succeeds in full motor execution, and the resulting neural activity. The robotic device, equipped with a digital signal processor, was used to guide the hand's motion, record the data of the hand movement, and synchronize with Flash^TM^ software for visual and auditory cues. Another robot device's purpose was to assist in returning the participant's hand to its original position after grasp movement and is a distinct process from the brain activity data in motor execution. The use of robotic devices provided the same level of assistance to all participants, ensuring consistency in data collection within the time limit of the experimental protocol. Each participant completed three runs of the active task, consisting of 14 trials per run, resulting in a total of 42 trials. The trials involved a grasp‐and‐pull task characteristic of the active motor task, where stroke patients exert maximum force during the palm grasp with their affected hand under therapist supervision, while the healthy control group performed the task using their non‐dominant hand.

Each trial followed this sequence: participants fixated their gaze on the monitor for 2 or 3 s, then performed each motor task for 2 s, following the visual and auditory cues. Participants maintained their grasping movement for 1 s before being instructed to release the grip handle. At the same time, the robotic device, controlled by a digital signal processor, had several functions, including storing the data of the executed hand movement, returning the participants’ hand to the initial position after the motor task was completed, and synchronizing with the Flash^TM^ software. This device was self‐produced and used for experimental purposes only. The Flash^TM^ software provided instructions to the participants and was connected to an EEG system (Active‐Two, Biosemi^TM^, Amsterdam, Netherlands). The detailed experimental design has been described in our previous studies (Kim et al. 2015; Park et al. 2015).

Generally, rapid motor recovery is not observed in the chronic phase, and the rate of recovery tends to decrease. Consequently, this study aimed to identify EEG characteristics associated with the actual motor function of stroke patients’ upper limb during voluntary movements, as measured by the FMA score and grip strength. It is important to clarify that although the FMA primarily quantifies motor recovery, this has a direct impact on functional ability after a stroke. The focus on motor function is essential, as improvements here are often a prerequisite for enhanced daily functioning. By measuring changes in motor ability, the FMA indirectly supports assessments of a patient's recovery trajectory and ability to perform daily tasks. Building on this, we examined the brain characteristics observed during the active task and explored whether these characteristics could serve as biomarkers for representing stroke patients’ motor function.

### 2.3 Analysis of EEG Signals

**FIGURE 1:**
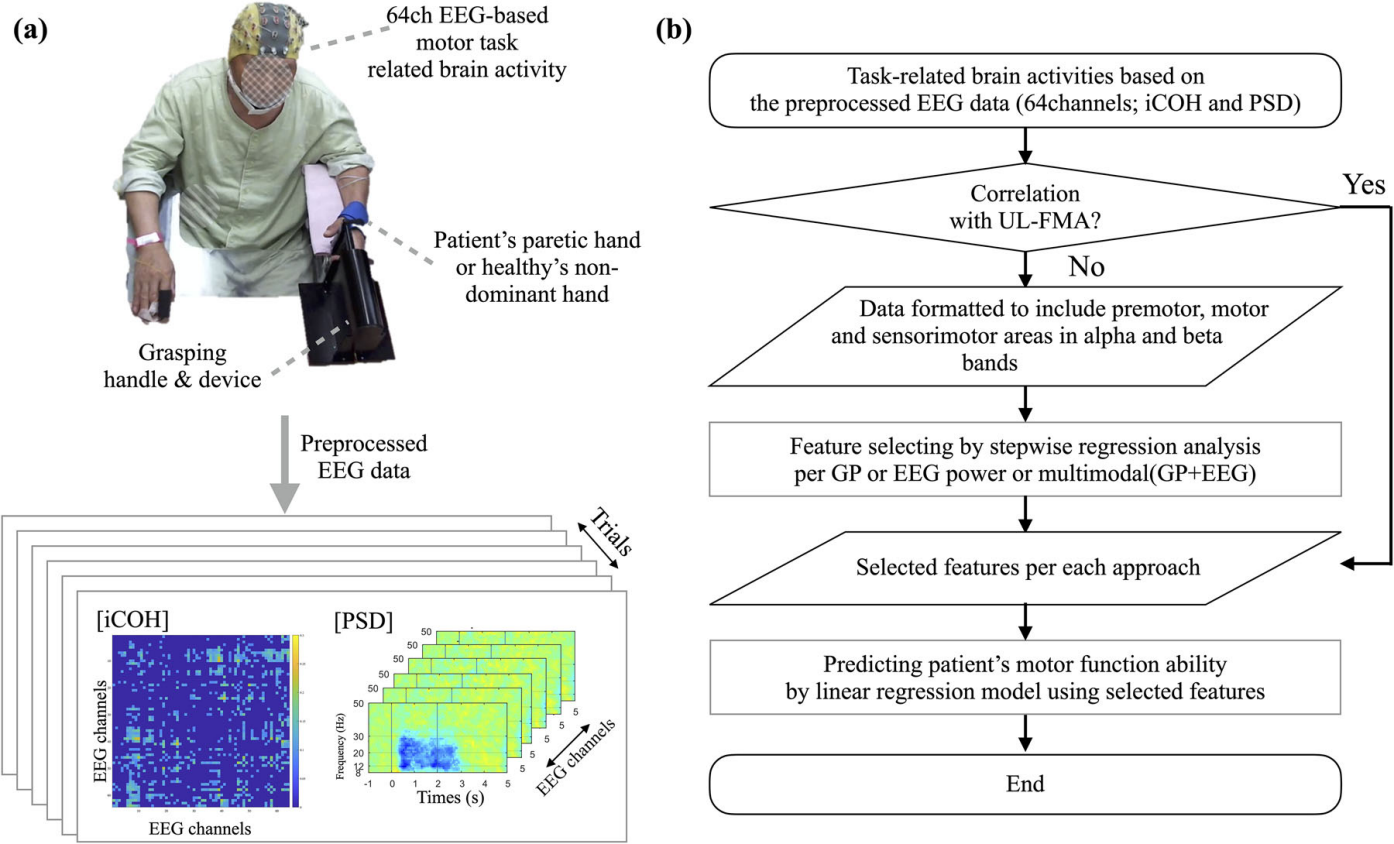
Experimental design and flow chart of EEG processing. (a) The Experimental design. When the visual event trigger is presented, the participant grasps their hand as hard as possible and after the task time, the robotic handle releases their hand to its initial state. After event extraction, the EEG data are pre‐processed according to the event trigger. The data is then transformed based on PSD and functional connectivity. The generated multimodal EEG features are selected through a stepwise regression analysis and then a regression model is generated to predict the patient's motor function ability. (b) Flow chart of EEG data processing. The research process consists of the following steps. The following process is to select features related to the patient's motor function based on task‐related brain activity based on iCOH and PSD characteristics. Then, a linear regression model based on these selected features can be built to determine the patient's motor performance. **Abbreviations**: EEG, electroencephalogram; iCOH, imagery of coherence; PSD, power spectrum density.

An outline of the EEG processing steps in this paper was shown in Figure 1(a). EEG signals were recorded at a sampling rate of 2,048 Hz using a 64‐channel active EEG electrode system. The EEG data were downsampled at 256 Hz and bandpass filtered at 1–80 Hz for preprocessing. The filtered EEG signals were then extracted into epochs between two seconds before the task cue and five seconds after the cue. The epoched EEG signals were then preprocessed by independent component analysis (ICA) to remove muscle artifacts (Vorobyov and Cichocki 2002) using the EEGlab toolbox (Delorme and Makeig 2004). The preprocessed EEG trials were then re‐referenced using the common average reference value to improve the quality of the signal‐to‐noise ratio (Nunez and Srinivasan 2006). To consider the symmetry of brain activity and the distribution of visualizations in general, the left hemisphere was set to be the contralateral hemisphere. In other words, if the patient's affected hand was the left hand, the EEG electrode data was flipped to set the left side of the brain to the contralateral hemisphere of the stroke data. Then, in the HC group, the EEG data was flipped when the motor task was performed by the non‐dominant hand (left hand), so the left side of the brain became the opposite hemisphere. This allowed all participants to have a consistent format of EEG characteristics according to the hand that performed the grasp movement. This approach minimizes bias and allows the characteristics according to the motor task to be generalized.

Subsequently, to obtain additional indices of functional impairment following stroke, we analyzed patients’ functional connectivity based on EEG signals (De Vico Fallani et al. 2014; Carter et al. 2012). We used the imaginary coherence component (iCOH) to analyze the functional connectivity using 64‐channel EEG electrodes (Nolte et al. 2004). In recent studies of EEG connectivity networks based on cortical sensor data, the issue of volume connections has emerged. This occurs because cortical sensors can inadvertently produce artificial similarities in brain source activities, leading to false interactions in the brain area (Schoffelen and Gross 2009). The iCOH is useful for sensor‐level functional connectivity to solve this problem by obtaining the imaginary part of the complex coherence (Guggisberg et al. 2019). In general, the cross‐spectral measure obtained from the signals x_i_(𝑡) and x_j_(𝑡), is given by:

$$S_{\mathrm{ij}}=\frac{1}{N}\sum X_{i}\left(f\right)X_{j}*\left(f\right)\left(N\mathrm{is}\;\mathrm{the}\;\mathrm{number}\;\mathrm{of}\;\mathrm{trials}\right)$$

where X_i_(𝑓) and X_j_(𝑓) are derived from Fourier transform of signals x_i_(𝑡) and x_j_(𝑡) per each epoch. Next, the complex‐coherence is as follows:

$$C_{\mathrm{ij}}\left(f\right)=ℜ\left\{C_{\mathrm{ij}}\left(f\right)\right\}+jℑ\left\{C_{\mathrm{ij}}\left(f\right)\right\}$$

The iCOH can be calculated directly either from the imaginary part of the complex coherence as follows:

$$iCOH\left(f\right)=ℑ\{C_{uv}\left(f\right)\}$$

Based on the above procedure, we computed the functional connectivity between all pairs of EEG channels, identifying the N × N relationships using iCOH (*N* is the number of EEG channels) per alpha and beta frequency band.

EEG data were extracted from −1 to 0 s before the onset of the pre‐motor task and from 0.25 to 1.25 s after the onset of the post‐motor task for the analysis of task‐related network characteristics. Previous studies have considered a movement preparation latency of 300 ms following cues for motor execution or motor imagery (Carrillo‐de‐la‐Peña et al. 2008; Xu et al. 2016). Furthermore, Patel et al. (2005) reported the utility of considering the response characteristics such as P300 and N200 in various EEG‐based neuroscience applications (Patel and Azzam 2005). In this study, we also considered the post‐stimulus response characteristics in our task cue. Hence, we set the time window for the post‐motor task while considering the motor response preparation after the cue. Additionally, our previous study demonstrated that this time window yielded meaningful EEG power and network characteristics in comparison to other time windows (Kim et al. 2015). Graph theory has previously been used to identify the structural and functional properties of brain networks (Philips et al. 2017; De Vico Fallani et al. 2014; Bullmore and Sporns 2009). In the present study, our aim was to identify meaningful features capable of detecting brain damage during upper limb movement. For this purpose, we utilized EEG power and conducted graph‐theoretic analysis to acquire information about the damaged brain network in the chronic phase of stroke (Bullmore and Sporns 2009). We analyzed the brain network using global and local graph theoretical parameters through the Brain Connectivity Toolbox, with the aim of elucidating which channels represent motor characteristics of functional connectivity related to stroke (Rubinov and Sporns 2010). Our analysis included EEG power and several local graph parameters, including degree centrality, local efficiency, and nodal clustering coefficient, as well as a global parameter such as global efficiency.

As a global (large‐scale) parameter, global efficiency is defined as the average inverse of the shortest path length. It serves as a fundamental measure of the functional integration of a network, reflecting both the brain's capacity to facilitate communication between regions and its tendency toward modularity (de Vico Fallani et al. 2009; De Vico Fallani et al. 2014). In this study, we utilized global efficiency as a threshold index to identify meaningful connections among the N x N functional brain connections of all pairs. The formula for global efficiency is as follows:

$$E\left(globalefficiency\right)=\frac{1}{n}\sum_{i\in N}E_{i}=\frac{1}{n}\sum_{i\in N}\frac{\sum_{j\in N\;j\neq i}d_{ij}^{-1}}{n-1}$$

Where E_i_ is the efficiency of node i, and d_ij_ is the shortest path length between node i and j.

In this study, the functional connectivity network was derived using an iCOH metric, which encompassed both pre‐motor and post‐motor tasks. Our goal was to extract meaningful connections characterized by high iCOH values, given that the iCOH metric encompasses all network connections. After arranging all pairs in descending order based on their iCOH values, we determined a threshold point where a significant difference in global efficiency between the stroke and HC groups was evident. This difference was observed in the range from the top 1% to the top 90% of connections for each frequency band when considering motor task‐related activity. While some researchers have determined threshold points for functional networks using network density, our approach focused on distinguishing different network characteristics between stroke patients and HC by using global efficiency as a representative measure of overall network integration and information processing. A detailed description of this procedure is presented in the statistical section.

Degree centrality, among the local (small‐scale) parameters, serves as the fundamental measure of node density, quantifying the number of connections associated with node i. Consequently, it plays a crucial role in identifying critical brain regions that function as hubs within the network. In this context, k_i_ denotes the degree of node *i*, while a_ij_ signifies the connection status between *i* and *j*.

$$k_{i}\;\left(degree\;centrality,DC\right)=\sum_{j\in N}a_{ij}$$

Local efficiency is a local parameter that assesses small‐worldness at the local network level, quantifying the informational efficiency of each node within its respective subnetwork. In this context, E_loc,i_ represents the local efficiency of node *i*, a_ij_ denotes the connection status between *i* and *j*, and d_jh_(N_i_) signifies the length of the shortest path between *j* and *h*, containing only neighbors of node *i*. By definition, local efficiency is affected by the shortest path length. Local efficiency provides a measure of the informational efficiency of each node within its designated subnetwork.

$$E_{loc,i}\left(local\;efficiency,LE\right)=\frac{\sum_{j,h\in N\;j\neq i}{\left(a_{ij}a_{ih}{\left[d_{jh}\left(N_{i}\right)\right]}^{-1}\right)}^{1/3}}{k_{i}\left(k_{i}-1\right)}$$

The nodal clustering coefficient is essentially the degree to which the node is connected to neighboring nodes and thus represents the tendency of nodes to cluster together and the modularity of node *i*. In the formula, the nodal clustering coefficient is only concerned with the connectivity between nodes connected to node *i*.

$$C_{i}\;\left(nodal\;clustering\;coefficient,CC\right)=\frac{2t_{i}}{k_{i}\left(k_{i}-1\right)}$$

C_i_ represents the clustering coefficient of node *i* (C_i_ = 0 for k_i _< 2), and $t_{i}$ stands for the number of triangles connected to node *i*, which serves as the fundamental measure of segregation.

Motor task‐related characteristics were assessed through the comparison of these graph‐theoretic parameters pre‐ and post‐motor tasks. Moreover, we calculated the ERD/S (event‐related de/synchronization; EEG power) by applying a short‐time Fourier transform to the preprocessed EEG data. ERD/S reflects the changes in a neuronal network induced by an endogenous Brain Computer Interface (BCI) paradigm. The pre‐motor task involved normalizing the ERD/S from −1 to 0 s, indicating the relative change during the motor task in comparison to the pre‐motor task. As a common method in EEG analysis, ERD/S signified task‐related brain activation. In this study, we extracted the ERD/S as the EEG power, which reflects the sensorimotor rhythm, for the purpose of comparing it with the graph parameters.

### 2.4 Statistics

In this study, task‐related activity was analyzed by comparing pre‐ and post‐motor task activity. The task‐related activity for the global/local brain parameters was determined as the relative value between the pre‐ and post‐task, as follows:

$$\mathrm{The}\;\mathrm{task}-\mathrm{related}\;\mathrm{activity}\;=\frac{\mathrm{post}\;\mathrm{task}-\mathrm{pre}\;\mathrm{task}}{\mathrm{pre}\;\mathrm{task}}\times 100$$

By using task‐related activity, we focused on functional connectivity (FC) that occurs exclusively during task performance by specifically analyzing brain activity during post‐motor task periods compared to pre‐motor task periods. As the absolute values of the data can vary depending on the subject and the environment, we normalized the EEG power and functional connectivity estimates recorded during post‐task performance to the estimates obtained at pre‐task. This normalization process reduces inter‐subject variability and allows a more precise focus on task‐induced changes in brain activity. This approach also helps us understand the changes that occur in the damaged brain during motor tasks. To verify the concept of such task‐related activity in terms of EEG power, we used ERD/S (event‐related desynchronization/synchronization) for EEG activation. By comparing the data from the task period to the rest period, we can more precisely identify the brain's functional responses to the task.

We used the Wilcoxon's rank‐sum test to analyze global and local graph parameters and identify differences in task‐related activity between the stroke and HC groups. Specifically, for a global graph parameter aimed at identifying the optimal threshold point that exhibited significantly different brain networks between the groups, we utilized global efficiency, which serves as an indicator of organizational information transfer within the entire brain network. Subsequently, within the context of local parameters, we extracted meaningful local graph parameters and EEG power features that displayed task‐related activity.

In order to select features that could be indicative of the patient's motor ability, we performed the following procedure (Figure 1(b)). First, to establish the correlation between local parameters and motor function, we conducted Spearman's correlation analysis. Through this systematic approach, we identified the specific cortical brain areas demonstrating significant task‐related activity. Second, we identified meaningful local parameters related to stroke motor function ability. Based on the significant local graph and EEG power features, we selected brain features closely associated with the stroke patient's motor function using linear stepwise regression (Kuhn and Johnson 2019). The selected features were re‐validated using leave‐one‐out bootstrapping, which is well documented in the literature as an effective method for dealing with small sample sizes (Tibshirani and Efron 1993; Stone 1974). To identify characteristics for the selected features, we performed an analysis of covariance (ANCOVA) to address the limitations of interpreting task‐related activity alone. ANCOVA accounts for pre‐task network characteristics as covariates, allowing us to evaluate group differences after statistically controlling for these covariates. This approach is beneficial in studies where pre‐task characteristics might differ between groups, such as in our S and HC groups (Huitema 2011). These selected features were subsequently applied to a linear regression model to evaluate their potential as a new biomarker for stroke motor function ability, specifically the patient's UL‐FMA score. The leave‐one‐out approach was employed for training and testing the regression analysis. The reliability of the regression analysis was assessed using root mean square error (RMSE), and adjusted R^2^ and the correlation between actual and estimated UL‐FMA scores (Kalra 2017). These elements were considered in evaluating the reliability of the regression analysis.

## 3 Results

### 3.1 Characteristics of the Brain Network

In this study, we identified the task‐related brain characteristics of global and local parameters by comparing between the pre‐ and post‐motor tasks.

#### 3.1.1 Global Parameter

**FIGURE 2:**
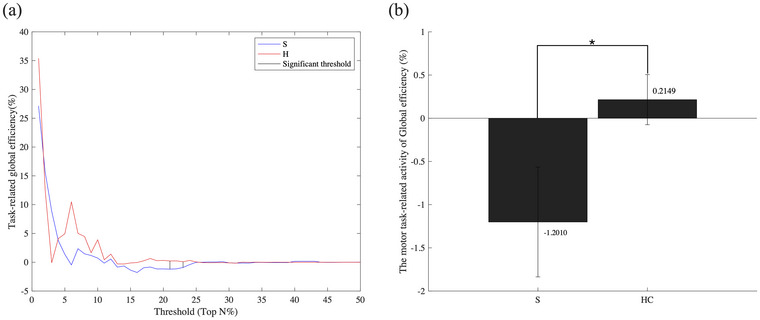
Motor task‐related changes in global efficiency. Task‐related global efficiency provides insight into the overall network properties and the impact of damage on connectivity. It was used to identify thresholds that distinguish the brain characteristics of stroke patients from healthy controls. (a) After the post‐motor task, the HC group showed an increase in global efficiency at most thresholds, whereas the stroke group showed a decrease. In particular, in the beta frequency band, meaningful thresholds for global functional connectivity emerged at 21 and 23% (p < 0.05). (b) Among these meaningful thresholds, the threshold at 21% represented the largest difference in task‐related global efficiency between the two groups. **Abbreviations**: S, stroke; HC, healthy control. * p < 0.05; Wilcoxon's rank‐sum test.

We analyzed global efficiency as a global parameter, quantifying the integration of the entire network and information flow throughout the brain network during the motor task (Sporns 2011). To identify the overall functional integration of the brain network, we employed this global parameter. Global parameters, such as global efficiency, offer valuable insights into the network's characteristics as a whole and meaningfully assess the impact of damage on network connectivity (Figure 2).

In the beta frequency band, we identified a significant threshold point for total functional connectivity at 21% (Wilcoxon's rank‐sum test, p < 0.05). Notably, while the HC group exhibited increased global efficiency between the pre‐ and post‐motor tasks, the stroke group displayed a decrease. This threshold revealed a statistically significant difference in task‐related global efficiency between the stroke and HC groups. Of these, the statistical difference of 21% (p‐value, 0.0175) was the largest, so this was chosen as the threshold value.

#### 3.1.2 Local Parameters

We identified the local characteristics using the local graph parameters, including degree centrality, which measures centrality, local efficiency for information processing, and nodal clustering coefficient, associated with modularity and network cost. These local graph parameters represent the within‐network communication during the motor execution task in the beta frequency band. Additionally, we compared the local graph patterns with the general EEG pattern by analyzing EEG power (ERD/S), which represents brain activation.

**FIGURE 3:**
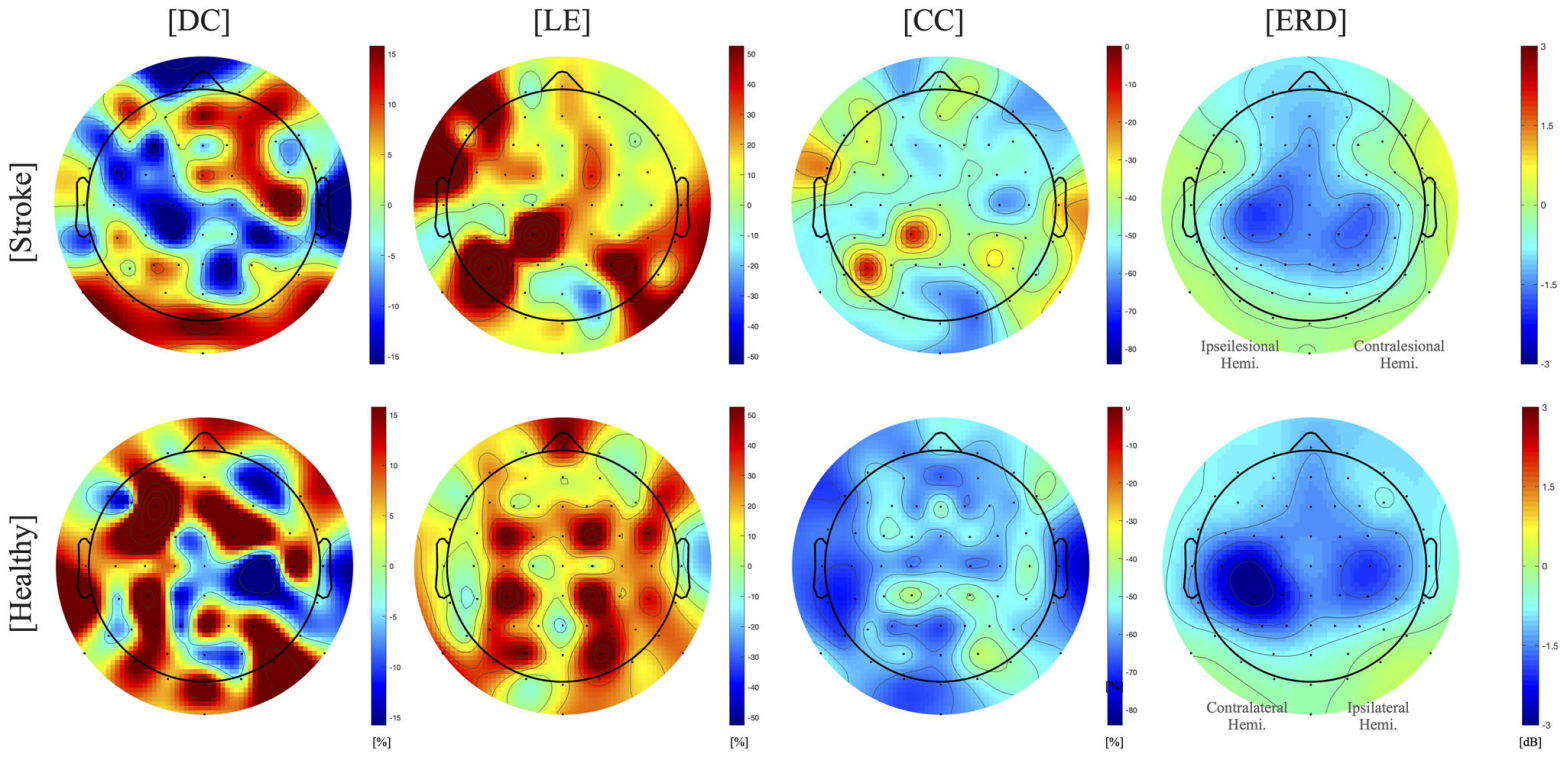
The significant difference between stroke and HC groups in the local parameters. In bilateral MA, only DC demonstrated significant differences between the stroke and HC groups, as evidenced by Wilcoxon's rank‐sum test (p < 0.05). While both stroke and HC groups showed comparable activation patterns in ERD, intriguingly, their network characteristics within bilateral MA diverged. The HC group, possessing an undamaged network, manifested a reduced network composition, attributed to the diminished role of the ipsilateral MA during motor tasks. In contrast, the stroke group presented a unique pattern, increasing DC in the contralesional MA while preserving functional segregation — reflected in LE and CC — to support the function of the ipsilesional MA. **Abbreviations**: HC, healthy controls; MA, motor area; DC, degree centrality; LE, local efficiency; CC, clustering coefficient; ERD, event‐related desynchronization.

In local parameters, as a quantitative interpretation, based on the difference between two groups (Wilcoxon's rank‐sum test < 0.05), only the degree centrality had significantly different patterns between the stroke and HC groups in bilateral MA (Figure 3). Although the HC group had increased centrality in the contralateral MA in the beta frequency band, the stroke group had decreased centrality (DC) in the ipsilesional MA (contralateral MA; C1) and slightly increased centrality in the contralesional MA (ipsilesional MA; C4).

The CC demonstrated a significant concentration in the contralateral MA. Furthermore, the characteristic of the CC displayed a decreasing trend in both groups, with a more pronounced decrease observed in the HC group, particularly in CP5 and Cz, when compared to the stroke group.

While LE and ERD did not exhibit statistically significant differences between the two groups, it was observed that the HC group displayed a more pronounced ERD and a decrease in task‐related LE compared to the stroke group, specifically within the bilateral MA.

Next, we performed a correlation analysis between each graph parameter in the MA and the patient's UL‐FMA score using Spearman's correlation in the beta frequency band. Most local graph parameters did not show significant correlations with stroke motor function, as measured by UL‐FMA scores using Spearman's correlation (p > 0.05). However, LE was correlated with an EEG channel (Cz)″.

Contrary to our initial expectations, we did not observe significant group differences or a correlation with the UL‐FMA in most of the local parameters. While the difference in global and local parameters between the stroke and HC groups during motor task performance was not statistically significant, it remained of interest due to their contrasting patterns across the two groups.

### 3.2 Estimations for Patient's Motor Function Abilities

#### 3.2.1 Feature Selection

Since the local characteristics showed no statistically significant differences between the stroke and HC groups and did not exhibit a significant correlation with UL‐FMA, we opted to utilize the local parameters of the bilateral MA (FC1, FC3, FC5, C1, C3, C5, CP1, CP3, CP5, FC2, FC4, FC6, C2, C4, C6, CP2, CP4, and CP6). The EEG channels of these bilateral MAs include the premotor area for planning movement, the primary motor area for executing movement, and the sensorimotor area for integrating sensory input and motor output. These parameters, commonly employed as biomarkers for stroke, were used as a feature in our regression analysis.

In our stepwise regression analysis, we employed the sum of squared errors for an F‐test as a criterion with a significance level of 0.05 to determine the addition or removal of variables. Additionally, we initialized the stepwise regression with a quadratic model, which included an intercept, linear terms, squared terms, and all possible products of pairs of predictors. Subsequently, guided by the criterion of the regression analysis, we identified the relevant features to be integrated into the regression model from the input elements. Consequently, the regression model utilizing local parameters demonstrated significant performance concerning EEG power, graph network parameters (GP), and their combination (referred to as ‘multimodal,’ comprising EEG power and network parameters) centered on the contralesional MA. These findings affirm the utility of contralesional MA's neural activity in relation to the patient's motor function, effectively serving as an estimator for the patient's motor status.

For each modality, our regression model was structured as follows:

$$\begin{array}{ccc} \mathrm{In}\;\mathrm{the}\;\mathrm{GP}\;\mathrm{model} & : & y∼1+\mathrm{DC}\left(C4\right)+\mathrm{LE}\left(\mathrm{CP}4\right)+\mathrm{CC}\left(C4\right)\\ & & +\;\mathrm{CC}\left(\mathrm{CP}4\right)+\mathrm{CC}\left(\mathrm{CP}2\right)+\mathrm{DC}{\left(C4\right)}^{2}+\mathrm{CC}{\left(C4\right)}^{2}\\ & & +\;\mathrm{CC}{\left(\mathrm{CP}4\right)}^{2}+\mathrm{CC}{\left(\mathrm{CP}2\right)}^{2} \end{array}$$

$$\begin{array}{ccc} \mathrm{In}\;\mathrm{the}\;\mathrm{ERD}\;\mathrm{model} & : & y∼1+C2+C4+C6+\mathrm{CP}4+\mathrm{CP}6+C4^{2}\\ & & +\;\mathrm{CP}4^{2}+C2*C4 \end{array}$$

$$\begin{array}{ccc} \mathrm{In}\;\mathrm{the}\;\mathrm{multimodal}\;\mathrm{model} & : & y∼1+\mathrm{CC}\left(C4\right)+\mathrm{ERD}\left(C2\right)\\ & & +\;\mathrm{ERD}\left(C4\right)+\mathrm{ERD}\left(C6\right)+\mathrm{ERD}\left(\mathrm{CP}4\right)\\ & & +\;\mathrm{ERD}\left(\mathrm{CP}6\right)+\mathrm{ERD}{\left(C4\right)}^{2}+\mathrm{ERD}{\left(\mathrm{CP}4\right)}^{2}\\ & & +\;\mathrm{ERD}\left(C2\right)*\mathrm{ERD}\left(C4\right) \end{array}$$

Table 2 presents the performance of the regression model for each EEG modality. The regression models of each modality all passed the F‐statistic test with statistical significance, and their adjusted R^2^ values closely approached 1. Furthermore, all the selected regression parameters demonstrated significant probability values, reinforcing the robustness of our analysis.

**TABLE 2:** The performance for each modal regression model.

		Unstandardized coefficients		standardized coefficients	t‐statistics	Probability value	Correlation coefficient	Fitted adj.R^2^	F‐statistics (p‐value)
		ß	Std.Error						
GP	DC (C4)	26.15	0.33	0.84	78.23	0.01	0.35	1.00	1.16e+04 (0.01)
	LE (CP4)	12.10	0.30	0.36	39.86	0.02	−0.05		
	CC (C4)	153.45	0.64	3.86	240.97	0.00	0.25		
	CC (CP4)	2.99	1.02	0.08	2.92	0.21	0.11		
	CC (CP2)	−72.88	0.66	−2.19	−110.30	0.01	−0.34		
	DC (C4)^2^	−35.40	0.50	−0.63	−70.54	0.01	−0.02		
	CC (C4)^2^	133.30	0.62	3.63	213.51	0.00	−0.25		
	CC (CP4)^2^	6.74	1.16	0.13	5.821	0.11	−0.11		
	CC (CP2)^2^	−49.77	0.61	−1.34	−81.19	0.01	0.34		
ERD	C2	44.44	4.33	5.310	10.27	0.01	−0.09	0.97	36.55 (0.03)
	C4	−75.73	7.52	−15.70	−10.07	0.01	−0.26		
	C6	83.19	8.62	17.31	9.65	0.01	0.10		
	CP6	−29.76	4.22	−5.83	−7.05	0.02	−0.12		
	CP4	−2.43	1.49	−0.44	−1.63	0.25	−0.47		
	C4^2^	−2.52	0.31	−2.28	−8.26	0.01	−0.06		
	CP4^2^	−13.42	1.53	−12.24	−8.79	0.01	0.31		
	C2*C4	19.03	2.00	11.68	9.50	0.01	0.26		
Multimodal (GP+ERD)	CC (C4)	5.73	0.01	0.14	822.26	0.00	0.26	1.00	6.78e+04 (9.24e‐19)
	C2	45.15	0.01	5.40	6023.85	0.00	−0.09		
	C4	−76.13	0.01	−15.78	−5879.95	0.00	−0.26		
	C6	86.91	0.02	18.09	5604.83	0.00	0.10		
	CP6	−33.58	0.01	−6.58	−3895.83	0.00	−0.12		
	CP4	−2.18	0.00	−0.40	−845.46	0.00	−0.47		
	C4^2^	−2.37	0.00	−2.14	−4239.29	0.00	−0.06		
	CP4^2^	−14.09	0.00	−12.85	−5126.46	0.00	0.31		
	C2*C4	19.68	0.00	12.08	5569.62	0.00	0.26		

Abbreviations: **adj.R^2^**, adjusted R^2^; **CC**, clustering coefficient; **DC**, degree centrality; **ERD**, event‐related desynchronization; **GP**, graph network parameter; **LE**, local efficiency.

In our regression model based on graph parameters derived from the contralesional MA, the selected features included DC(C4), LE (CP4), CC(C4), CC(CP4), DC(C4)^2^, CC(C4)^2^, CC(CP4)^2^, and CC(CP2)^2^. Subsequently, we conducted a regression analysis using EEG power as a control method. Our EEG power regression model incorporated features from C2, C4, C6, CP4, and CP6. However, it is worth noting that the EEG power model yielded a slightly lower adjusted R^2^ value and a large standard error compared to the GP model. Finally, in the case of the multimodal model, which combines both modalities, it demonstrated significantly superior performance compared to the unimodal models. Interestingly, we also introduced the factor of CC(C4) into the EEG power model, resulting in a substantial reduction in the standard error and a low probability value. Both unimodal and multimodal regression models, whether based on GP or EEG power, exhibited commendable performance when tailored to the characteristics of the contralesional MA.

To provide evidence of the validity of the features selected in this study, we performed a bootstrapping analysis with 1000 runs, using a leave‐one‐out approach during the feature selection process for the multimodal model. This eliminated the risk of overfitting and ensured that the features were selected equally in our study. As a result, the bootstrapping analysis consistently selected features related to the contralesional motor area, particularly CC (C4) and EEG power features (C2, C4, C6, CP6, and CP4). These features appeared with high probability, confirming their reliability as prognostic indicators.

#### 3.2.2 Estimating Motor Function Abilities Using Linear Regression

We performed linear regression analysis based on each estimation regression equation using a leave‐one‐out approach (Table 3). Additionally, we performed a reliability test for each regression analysis using RMSE, adjusted R^2^, and the correlation between the actual and predicted UL‐FMA score.

**TABLE 3:** The predicted results for UL‐FMA score using leave‐one‐out approach.

Patients			S1	S2	S3	S4	S5	S6	S7	S8	S9	S10	S11	RMSE	R^2^ /adj.R^2^	F‐statics (p‐value)	Correlation
Chronic phase	Actual UL‐FMA		31	56	44	52	32	40	54	48	45	59	63				
	Predicted UL‐FMA	GP	30.34	54.23	44.16	51.73	32.58	42.85	52.79	48.74	44.82	64.99	63.44	2.24	0.96 /0.95	230 (1.02e‐07)	0.9810
		ERD	46.07	55.46	48.66	55.16	21.08	34.44	54.36	48.35	59.28	55.21	66.70	8.15	0.61 /0.57	14.5 (0.00415)	0.7857
		Multimodal (GP+ERD)	31.45	56.02	44.09	51.96	32.03	39.98	54.01	47.99	45.04	59.14	62.99	0.13	0.99 /0.99	6.78e+04 (9.24e‐19)	0.9999
Early chronic phase	Actual UL‐FMA		51	66	66	36	61	55	21	46	14	66					
	Predicted UL‐FMA	GP	41.60	101.50	59.05	25.57	67.12	71.09	18.49	62.93	21.78	62.88		15.1	0.70 /0.67	18.8 (0.00248)	0.8378
		ERD	50.35	110.40	61.33	35.85	61.06	54.89	20.73	46.52	14.14	65.71		14.5	0.74 /0.71	22.7 (0.00142)	0.8599
		Multimodal (GP+ERD)	44.48	73.95	75.53	39.70	60.64	55.40	22.59	42.28	12.73	68.59		4.85	0.95 /0.94	164 (1.3e‐06)	0.9765

Abbreviation: **adj.R^2^**, adjusted R^2^; **ERD**, event‐related desynchronization; **GP**, graph network parameter; **RMSE**, root mean squared error; **UL‐FMA**, upper limb Fugl‐Meyer assessment.

In this study, the ERD regression model exhibited suboptimal performance (adjusted R^2^ = 0.57, RMSE = 8.15), while the GP model demonstrated relatively robust performance (adjusted R^2^ = 0.95, RMSE = 2.24). Remarkably, the multimodal regression model achieved the highest level of performance, boasting an adjusted R^2^ value of 0.99 and an RMSE of 0.13, even after undergoing cross‐validation against a comprehensive full‐participant model. While the adjusted R^2^ values obtained from the stepwise regression analysis for feature selection approach 1 for all modalities, it is important to note that in the cross‐validated full‐participant estimation model using the leave‐one‐out approach, sustained high performance was observed only in the GP and multimodal models (Table 3). This finding reports the important role of the graph parameters and the absence of overfitting problems. It is also evident that the best performing multimodal model had a smaller standard error of prognostic factors compared to the other unimodal regression model (Table 2, standard error < 0.2).

For external validation for this model of chronic phase, we constructed a regression model of the early chronic phase dataset based on the contralesional MA feature. This model resulted in the confirmation of significant performance in the GP+ERD model. Although this regression results of the early chronic phase dataset were statistically significant, RMSE was somewhat larger than that of the existing chronic phase dataset (RMSE 4.85/ adj.R^2^ 0.948).

#### 3.2.3 The Characteristics of the Contralesional Motor Area in Stroke Patients

We evaluated the key regression factors, as outlined in Table 2, and assessed their impact within the regression model by analyzing their correlations with UL‐FMA scores and examining their standardized coefficients. In addition to the EEG power of the contralesional MA, CC, and DC played pivotal roles in the regression model. To identify detailed characteristics of C4, we performed an ANCOVA analysis to obtain additional information related to task‐related activity. ANCOVA analysis accounts for pre‐task network characteristics as covariates, thereby enabling the evaluation of group differences after statistically controlling for these covariates. The ANCOVA analysis conducted across all EEG channels for each network parameter confirmed significant differences between the S and HC groups in the C4 area for both DC and CC (p < 0.05).

**FIGURE 4:**
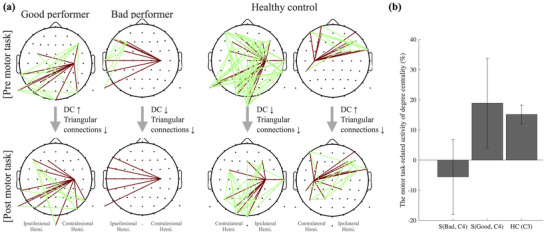
A comparison of network characteristics among HCs and stroke subgroups based on their motor function performance. (a) Connection plot in the pre‐ and post‐motor tasks. The topoplot illustrates DC connections of C4 in brown and triangular connections within EEG channels linked to C4 in green (related with CC). For the HC group, both DC and CC connections observed notable reductions transitioning from pre‐ to post‐motor tasks, particularly centered on long‐distance connections. Similarly, reductions in both DC and CC were evident in stroke patients. Intriguingly, among these stroke patients, those who performed motor tasks poorly (UL‐FMA score range 31–47) demonstrated more pronounced declines in both DC and CC compared to good performers (UL‐FMA score range 48–61). This observation underscores the significant role of the contralesional MA. It aligns with the findings of our regression model, which indicate that stroke patients with a more preserved network surrounding the contralesional MA tend to exhibit better motor performance. (b) The motor task‐related DCs among subgroups. The bad performers (UL‐FMA score range 31–47) in the stroke group showed a pattern of decreasing task‐related DCs in the contralesional M1 (C4), while the good performers (UL‐FMA score range 48–61) and the HC group in the contralateral M1 (C3) showed a pattern of increasing DCs. This represents that the greater the similarity between the contralesional M1 in the stroke group and the characteristics of the contralateral M1 in the HC group, the better the motor function in the stroke group.

To identify detailed differences on C4, we conducted Wilcoxon's rank sum tests to compare pre‐task and post‐task network characteristics between the groups (Table 4). As a result, HC showed a decrease in both DC and CC at post‐task compared to pre‐task, while S showed a slight increase in DC and a lesser decrease in CC. These characteristics were similar to the changes in network characteristics of HC in C3. The H group, which had a normal network without lesions, showed, on average, a slight increase in the number of connections (DC) and a decrease in the clustering properties (triangle connections) of connected regions in C3 during post‐task (contralateral M1) (Figure 4 (a)).

**TABLE 4:** Comparisons for network pattern changes in the bilateral M1.

			Wilcoxon's rank sum test			
			Pre‐task	Post‐task	Task‐related activity (%)	ANCOVA (F‐static/p‐value)
C4	DC	Mean of S	11.90	12.20	0.56	4.98 (0.0192)
		Mean of HC	12	7.20	−34.04	
		p‐value	1	0.0338	0.0290	
	CC	Mean of S	0.0880	0.0270	−64.12	4.51 (0.0268)
		Mean of HC	0.0597	0.0252	−50.74	
		p‐value	0.0452	0.8798	0.5966	
C3	DC	Mean of S	8.10	6.90	−13.94	1.29 (0.297)
		Mean of HC	16.8182	17.9091	3.10	
		p‐value	0.0309	0.0236	0.2744	
	CC	Mean of S	0.0837	0.0503	−57.40	8.23 (0.0027)
		Mean of HC	0.0585	0.0220	−61.53	
		p‐value	0.0356	0.0660	0.5035	

Abbreviation: **ANCOVA**, analysis of covariance; **CC**, clustering coefficient; **DC**, degree centrality; **M1**, primary motor cortex.

Similar to the characteristics on C3 of the HC group, when a stronger ERD is observed in the C4 region, along with less destroyed modularity (CC), and the less diminished the DC, the patient's motor function performance is more favorable. These combined factors collectively contribute to the estimation of the patient's performance in the UL‐FMA (Table 2, the correlation coefficient). Figure 4(a) illustrates the connections (DC) of C4 and the modularity (CC) within the channels connected to C4 during both the pre‐ and post‐motor tasks. This allows us to observe how network characteristics change before and after the motor task. The results suggest that, in comparison to the pre‐motor task, individuals with good motor performance in the chronic phase of stroke displayed an increase in degree centrality (DC) and less pronounced decreases in clustering coefficient (CC) following the motor task, in contrast to those with poor performance. A correlation was observed between the aforementioned characteristics and the FMA score. While not statistically significant, a greater increase in the stroke's DC and a lesser change in CC in contralesional M1 were found to be positively correlated with the FMA score (Spearman's correlation; r = 0.47, p = 0.14). In other words, the more similar the C4 (contralesional M1) in the stroke group was to the characteristics of the contralateral MA in the HC group, the better the motor function of the stroke was. Our findings also indicated that the more the network characteristic changed in C4, the more contralesional M1 of the stroke group was similar to those in the contralateral M1 of the HC group, the better the patient's motor function. This was particularly evident in DC, where the network characteristic differences between groups were significant at post‐task (Figure 4(b)). As shown in the bar graph, the bad performers (UL‐FMA score range 31–47) in the stroke group showed a pattern of decreasing task‐related DCs in C4, while the good performers (UL‐FMA score range 48–61) and the HC group in C3 showed a pattern of increasing DCs.

## 4 Discussion

Our study aimed to explore the neurophysiological characteristics of brain damage in stroke patients and assess their potential utility in estimating UL‐FMA scores. Moreover, we sought to investigate task‐related brain responses during motor tasks to underscore the need for biomarkers in evaluating patients’ motor function. Notably, our results revealed significant disparities in global and local brain characteristics between stroke patients and HC, and we employed these distinctive brain activity patterns to estimate motor function ability in stroke patients.

### 4.1 Stroke's Brain Characteristics Compared With HC Group

Previous studies have shown that during motor tasks, network configurations transiently change, favoring a more globally efficient and less clustered network for more efficient information transmission (Di et al. 2013). In addition, the HC group would exhibit a more efficient network in comparison to stroke participants, as suggested by previous studies (de Vico Fallani et al. 2009; Lee et al. 2018). In alignment with previous studies, our results highlight distinct network characteristics between the HC group and stroke patients. Specifically, brain networks in stroke patients during motor tasks are less efficient compared to the HC group (de Vico Fallani et al. 2009; Di et al. 2013; Cheng et al. 2021). While Cheng et al. (2021) focused on analyzing functional connectivity strength in stroke patients across different motor tasks, the aforementioned studies collectively underscore the inefficiency of brain network functioning in stroke during motor tasks relative to the HC group. Such diminished global efficiency in the stroke group arises from disrupted connections and impaired information processing due to brain injuries, as highlighted in various studies (de Vico Fallani et al. 2009; Jiang et al. 2013; Di et al. 2013; Snyder et al. 2021). Guggisberg et al. (2019) also emphasized this point, suggesting that stroke patients lose the ability to efficiently transmit information due to modular fragmentation and weak inter‐modular integration in the injured brain (Guggisberg et al. 2019). The global network characteristics of the results of this study and previous studies suggest that the composition of the network responsible for performing motor tasks in stroke patients is different from that of HCs due to the damaged brain. Therefore, it is necessary to identify the network reorganization for the recovery of motor function in patients (López‐Larraz et al. 2018).

The results of the present study suggested that HC and stroke had opposite local characteristics in bilateral MA (Figure 3). Our results are consistent with different network characteristics in information processing between the ipsilesional and contralesional hemispheres due to the damaged brain caused by stroke (Guggisberg et al. 2019; Alia et al. 2017). Our study identified more specific brain network characteristics related to functional compensatory effects using graph parameters, despite the stroke and HC groups exhibiting similar brain activation patterns in bilateral MAs in ERD. Following the characterization of the common regression feature, C4 (Table 4), it was observed that the change in network characteristics between pre‐ and post‐task for the stroke was more clearly similar to the contralateral MA of the HC group than in Figure 2. Among the stroke group, those who exhibited better performance had network characteristics of their contralesional M1 for the stroke (C4) that were similar to those of the contralateral M1 for the HC (C3) characteristics of the HC group. This finding is consistent with that of a previous study by Storti et al. (2016), which demonstrated that the HC group exhibited increased centrality in the contralateral MA during motor task performance (Storti et al. 2016). On the other hand, the ipsilateral MA of HC showed a lack of local changes due to its inhibitory properties during motor task performance in the non‐damaged brain (Takeuchi et al. 2012). In alignment with these previous studies, while in this study the HC group exhibited increased DC and decreased CC in the contralateral MA than in the ipsilateral MA in this study, stroke patients showed these characteristics in the contralesional (ipsilateral) MA. Also, for these network characteristics for the stroke, we assumed that the peripheral MA was overconnected to activate the abnormal network owing to the increased cost of performing a task after a stroke (Hillary and Grafman 2017). Consistent with our assumption, stroke patients had contralesional network characteristics. Previous results support the network characteristics of stroke patients observed in this study. Cramer et al. showed that there is overactivation of the contralesional MA to compensate for the damaged region, occurring through increased cost by activating the peripheral MA following a stroke in an fMRI study (Cramer et al. 1997). Similarly, Biernaskie argued that dendritic growth in the undamaged MA forms a network in the contralesional hemisphere via adaptive plasticity (Cramer et al. 1997). These previous studies indicate that strokes lead to significant brain activation in the bilateral MA because of compensatory effects (Cramer et al. 1997; Biernaskie and Corbett 2001). The bilateral network characteristics in this study represented the compensatory effect depending on whether the stroke had abnormal information propagation compared to HC, similar to previous studies. They showed different network characteristics between the ipsilateral and contralateral hemispheres for information processing (Guggisberg et al. 2019, Alia et al. 2017). The current study found more specific brain network characteristics regarding functional compensatory effects using graph parameters than previous studies, although the stroke and HC groups had similar brain activation patterns in the bilateral MA in ERD patterns. In this study, the stroke group had lower degree centrality and local efficiency centered in the ipsilesional MA and higher degree centrality in the contralesional MA than the HC group. Likewise, de Vico Fallani et al. (2009) reported that results from a stroke do not lead to normal information propagation compared to the HC group using global and local efficiency (de Vico Fallani et al. 2009). In addition, Jiang et al. (Jiang et al. 2013) reviewed that stroke activates the brain area for motor tasks and has a low efficiency by increasing the disconnected link to overcome functional deficits of the motor pathway in the damaged brain.

Although the previous studies reported mostly global network characteristics, the bilateral local network properties for the stroke in this study, which had different characteristics from the HC, might be another piece of evidence of the damaged brain network by stroke lesion, such as findings in previous studies. These opposing characteristics observed in stroke patients compared to the HC group in terms of local network parameters suggest that the motor task‐related network is hindered by suboptimal path formation due to the lesion. This also suggests that contralesional MA may form networks to replace the role of ipsilesional MA. Moreover, in the next section in this study, we were able to estimate a reliable UL‐FMA based on the brain properties of contralesional MA (Table 2).

### 4.2 The Role of the Contralesional MA

Several studies have consistently emphasized the contralesional MA's significance in stroke research, especially its notable correlation with patients’ motor function and their FMA scores (Philips et al. 2017; Buetefisch 2015; Shim et al. 2023). Buetefisch (2015) reported that brain activity in the contralesional MA influences the improvement of motor function ability for the patient's paretic limb (Buetefisch 2015). Philips et al. (2017) found that local efficiency in the unaffected hemisphere is correlated with stroke motor function ability (Philips et al. 2017). Similarly, Shim et al. (2023) demonstrated that the contralesional area is efficient compared to the ipsilesional area (Shim et al. 2023). While our findings did not pinpoint a direct correlation between the local properties of contralesional MA and UL‐FMA scores, we reported the importance of these local parameters in estimating the motor function of stroke patients. Furthermore, in the early chronic phase dataset for external validation, the regression model consisting of the contralesional MA GP+ERD as a feature also demonstrated robust performance, as seen in the existing chronic phase dataset. This finding suggests that the outcomes of our existing research are valid despite the limitations of the sample size and is significant in that it provides substantial evidence that contralesional MA can serve as a reliable indicator of motor function in chronic patients. Conversely, the properties of the ipsilesional MA were not identified as key features for motor function in the stepwise regression analysis.

This was especially evident when focusing on connectivity alterations in the contralesional MA, specifically C4, which was primarily considered a prognostic factor (Figure 4(a)). The results revealed significant reductions in both DC and CC in the ipsilateral MA for the HC group, consistent with typical brain characteristics. In an undamaged brain, these changes could be interpreted as characteristics related to inter‐hemispheric interaction inhibition in the ipsilateral MA during motor task performance (Takeuchi et al. 2012). In contrast, stroke patients exhibited a smaller number of connections for both pre‐ and post‐motor tasks compared to the HC group. Those with poor performance exhibited particularly notable small numbers of connections for those with bad performers, resulting in correspondingly smaller pre‐ and post‐task changes. Furthermore, individuals who performed better with higher UL‐FMA scores were characterized by stronger ERD, less reduction in CC, and an increase in DC in the contralesional MA (Table 2, Figure 4). This suggests that, unlike the HC group, where the contralesional MA does not play a primary role in motor task performance, stroke patients engage in network construction to support or replace the function of the ipsilesional MA. In particular, better performers increased DC and did not deconstruct modularity (CC) as much as possible. These characteristics suggest that the preservation of clustered subnetwork formation during task‐related network construction, allowing for the fulfillment of alternative roles, may have an influence on motor function performance (Guggisberg et al. 2019).

These results provide evidence for a potential alternative role of the contralesional MA in the chronic phase of stroke. Expanding upon the findings of our study and previous research regarding the potential role of the contralesional MA, it is conceivable that the features of these local parameters in the contralesional hemisphere may influence the estimation models of patients' motor function. While the exact role of the contralesional MA remains debated in spite of their functional changes (Alia et al. 2017; Shim et al. 2023), Guggisberg et al. (2019) reviewed its undeniable influence on functional recovery, even amid conflicting views on its exact contribution during stroke recovery (Guggisberg et al. 2019). Accordingly, Paul et al. (2021) suggest that these conflicting results on the role of contralesional MA may be attributed to the specific motor tasks performed in each study (Paul et al. 2021). Additionally, Volz et al. (2017) investigated the role of contralesional MA in stroke by restricting its activity using transcranial magnetic stimulation (TMS) during the subacute and chronic phases, respectively. They found that it improved motor performance in the subacute phase but did not affect performance in the chronic phase. These findings suggest that the properties of the contralesional MA in stroke may have time‐dependent characteristics, and that activity inhibition in this region may not affect motor performance in the chronic phase (Volz et al. 2017). Our findings further amplify the potential of contralesional MA's brain properties as indicative biomarkers for motor function.

### 4.3 Multimodal Biomarkers for Stroke Motor Function Abilities

Incorporating a multimodal approach, our study pinpointed a model for motor function estimation based on brain activity in the contralesional MA that surpassed unimodal models. Specifically, the multimodal model demonstrated an impressive adj. R^2^ value of 0.99 with an RMSE of 0.13, revealing the usefulness of assessing brain network characteristics through graph parameters in motor function estimation. In particular, maintaining subnetworks in the contralesional MA in a supportive role had a positive impact on predicting motor function during motor task performance in stroke patients in the chronic phase, where alternative pathways had become established. These results indicate the importance of assessing brain network characteristics through graph parameters, rather than just relying on brain activation, when estimating patients' motor function. While it may be considered unreasonable to use only graph parameters, graph properties are employed to quantify the organization and efficiency of networks related to motor recovery (de Vico Fallani et al. 2009; Jiang et al. 2013; Guggisberg et al. 2019; Hillary and Grafman 2017, Vecchio et al. 2025). These graph properties can evaluate network‐level interactions that are difficult to observe with ERD/S patterns. The findings of this study suggest that graph properties can play a significant role in providing insights into the patient's condition.

Our study reported more reliable biomarkers compared to previous studies focused on EEG activity biomarkers for chronic stroke. In a motor execution task, Zhang et al. (2019) predicted FMA scores using EEG power‐based deep learning during the button‐clicking movement (cross‐participants test, 14 chronic strokes, R^2^ 0.47) (Zhang et al. 2019). During the EEG resting state, Riahi et al. (2020) reported a high regression coefficient for predicting FMA scores using EEG‐based contralesional connectivity features (10 chronic strokes, R^2^ 0.91) (Riahi et al. 2020). Sun et al. (2021) regressed the FMA score using EEG connectivity strength in the resting state after robot‐assisted training (12 chronic strokes, R^2^ 0.81) (Sun et al. 2021). In comparison, our study reported a high regression coefficient for EEG‐based network features during voluntary hand movements (10 chronic strokes, R^2^ 0.99). While previous studies regressed FMA scores using mostly resting‐state EEG data, we used EEG task‐related activity during hand movement in stroke patients to get better performance. Our study indicates the importance of identifying task‐related biomarkers and predictors for estimating patients’ motor function rather than relying just on physical features or the resting state of the damaged brain.

While our study represents a significant advancement in the field of EEG‐based biomarkers for chronic stroke, it's important to acknowledge its limitations. The small sample size and exclusion of severely impaired cases mean our findings, though promising, may not be universally applicable. Although we did not include severely impaired stroke patients, our findings may have significance for individuals with mild to moderate impairments, especially considering that rehabilitation goals are tailored to the patient's condition (Langhorne et al. 2011; Lang et al. 2013). Furthermore, our study focused on brain activity characteristics occurring during voluntary movement, making it particularly relevant for mild to moderate stroke cases where hand movements are possible. Our characteristic on the contralesional MA can support the patient's rehabilitation program by guiding the chronic brain network that can be linked with the whole brain to enhance the patient's motor recovery. It may provide a biomarker to monitor patients' status and adjust treatment accordingly during regular hospital visits. And since it's an EEG‐based biomarker, it's a relatively inexpensive way to determine a patient's condition, which should have an economic impact.

## 5 Conclusions

Our study has provided valuable insights into the neurophysiological characteristics of stroke patients and their potential implications for estimating UL‐FMA scores. Through our research, we aimed to explore the distinct neurophysiological traits of stroke patients in comparison to HC and their relevance in assessing motor function.

Notably, stroke patients exhibited lower task‐related global efficiency, reflecting less efficient information processing, which can be attributed to the impact of brain injury. This underscores that stroke patients had different network properties responsible for motor tasks in the damaged brain in comparison with HC. Furthermore, our investigation into local network characteristics, with a focus on bilateral MAs, has revealed distinctive patterns in stroke patients compared to HC. These distinctions emphasize the influence of stroke‐induced brain damage on information processing within both the ipsilesional and contralesional hemispheres. Particularly noteworthy is the contralesional MA, which exhibited unique network features associated with functional compensatory effects, suggesting its potential role in supporting or even substituting the function of the ipsilesional MA.

Our research revealed three key findings:

Stroke patients exhibited lower task‐related global efficiency compared to HC, reflecting less efficient information processing due to brain injury.

Stroke patients with contralesional motor areas (MAs) that exhibited characteristics similar to the contralateral MAs of HC demonstrated better motor function.

The unique network features of the contralesional MA are crucial for predicting patient status, highlighting its role in reflecting connectivity disruptions caused by brain damage.

These findings underscore the different network properties responsible for motor tasks in the damaged brains of stroke patients compared to HCs. Also, the investigation into local network characteristics, focusing on bilateral MAs, revealed distinctive patterns in stroke patients. These patterns emphasized the compensatory effect in stroke‐induced brain damage for information processing within the contralesional MA.

Our study adopted a multimodal approach, incorporating both brain activity and network characteristics, which outperformed unimodal models. This highlights the importance of assessing brain network properties through graph parameters when estimating motor function in stroke patients.

In summary, the brain characteristics of contralesional MA in patients with early chronic/chronic phase stroke contribute to understanding the neurophysiological aspects of stroke‐related motor impairments and suggest the possibility of using it as a meaningful biomarker to help assess the progress of therapy and the development of more effective rehabilitation strategies in the chronic phase.

## Author Contributions

DHK participated in designing the study, performed statistical analysis, and wrote the manuscript. GHK participated in experimental design, selecting the clinical population, and adapting the protocol for patients. SWL and LK conceived and coordinated the study and helped draft the manuscript. All authors have read and approved the final version of the manuscript.

### Peer Review

The peer review history for this article is available at https://publons.com/publon/10.1002/brb3.70492

## Data Availability

The EEG data that support the findings of this study are available on request from the corresponding author for the research purposes. The data are not publicly available due to their containing information that could compromise the privacy of research participants.
